# Glaucoma detection using non-perfused areas in OCTA

**DOI:** 10.1038/s41598-024-60839-4

**Published:** 2024-05-05

**Authors:** Julia Schottenhamml, Tobias Würfl, Stefan Ploner, Lennart Husvogt, Robert Lämmer, Bettina Hohberger, Andreas Maier, Christian Mardin

**Affiliations:** 1 Department of Ophthalmology, Universitätsklinikum Erlangen, Friedrich-Alexander-Universität Erlangen, Nürnberg, Erlangen Germany; 2 Pattern Recognition Lab, Friedrich-Alexander-Universität Erlangen, Nürnberg, Erlangen Germany

**Keywords:** Diagnostic markers, Computer science, Glaucoma, Diagnostic markers

## Abstract

Multiple ophthalmic diseases lead to decreased capillary perfusion that can be visualized using optical coherence tomography angiography images. To quantify the decrease in perfusion, past studies have often used the vessel density, which is the percentage of vessel pixels in the image. However, this method is often not sensitive enough to detect subtle changes in early pathology. More recent methods are based on quantifying non-perfused or intercapillary areas between the vessels. These methods rely upon the accuracy of vessel segmentation, which is a challenging task and therefore a limiting factor for reliability. Intercapillary areas computed from perfusion-distance measures are less sensitive to errors in the vessel segmentation since the distance to the next vessel is only slightly changing if gaps are present in the segmentation. We present a novel method for distinguishing between glaucoma patients and healthy controls based on features computed from the probability density function of these perfusion-distance areas. The proposed approach is evaluated on different capillary plexuses and outperforms previously proposed methods that use handcrafted features for classification. Moreover the results of the proposed method are in the same range as the ones of convolutional neural networks trained on the raw input images and is therefore a computationally efficient, simple to implement and explainable alternative to deep learning-based approaches.

## Introduction

Optical coherence tomography (OCT)^1^ is a non-invasive, three-dimensional imaging technique that allows in-vivo visualization of tissue on a micrometer scale. OCT angiography (OCTA), an extension of OCT, can display blood vessels by visualizing signal changes caused by moving particles, in this case erythrocytes in the blood. Since OCTA is computed from OCT scans, it shares the same positive properties as OCT as an imaging technique. Because of these characteristics, OCT and OCTA are in widespread use in ophthalmic settings, where their depth resolution enables separate analysis of the retinal plexuses. This enables new possibilities to study disease pathogenesis and progression and aid in the diagnosis, since previous ophthalmic imaging modalities were limited to two-dimensional images, where deeper plexuses are either merged with more superficial ones, or not visible at all.

An eye disease that attracts continued attention in ophthalmology is glaucoma. This group of neurodegenerative eye diseases manifests by a degeneration of retinal ganglion cells in the peripapillary retinal nerve fibre layer and specific alterations of the optic disc. Glaucoma is among the leading causes of irreversible blindness worldwide^2^. However, the onset and progression can be slowed down or even stopped if diagnosed and treated early enough. Up to this date, the pathogenesis of glaucoma is not completely understood. Since glaucoma manifests in these structural changes of the retinal tissue, most of the research focuses on detection and quantification of structural alterations in OCT scans. Recent studies found a link between the onset and progression of glaucoma and reduced ocular blood flow^3–5^.

This reduced blood flow can be visualized using OCTA. A widely used biomarker is the vessel density (VD), which has been extensively studied in different regions and plexuses with varying degrees of success^6–12^. Schottenhamml et al.^13^ demonstrated that it is possible to use small $3 \times 3~\hbox {mm}$ macular scans to distinguish glaucoma patients and healthy controls. They showed that deep learning methods can achieve state-of-the-art performance and outperform classical features like the vessel density. However, deep learning methods are black-box approaches and the features computed by neural networks are challenging to interpret for humans. In order to study the areas that the network uses to detect glaucoma, the authors used attention maps^14^ to highlight areas in the image that influence the decision of the network for disease classification. Their visual results indicated that the network concentrates on areas with a reduced perfusion. This observation implies that these regions are a very good biomarker for detecting glaucoma but that the VD is not sensitive enough to detect smaller changes.

A more sensitive feature that can be used to measure reduced vascular perfusion are non-perfused or intercapillary areas which have been investigated in other retinal diseases^15–17^. These approaches quantify the areas between the vessels. This is assumed to be more sensitive since even if only some small vessels are not visible on OCTA images anymore, the area of these non-perfused areas can change notably, while the vessel density will not be influenced as much. However, in order to quantify intercapillary areas, a very accurate segmentation of the vascular network is needed, because small errors and gaps in the segmentation will affect the results in an equally strong manner.

A more robust alternative to using intercapillary areas is the usage of the perfusion distance. This approach computes the distance from any pixel to its next vessel pixel and is consequently not as sensitive to smaller errors in the vessel segmentation. Lauermann et al.^18^ found a statistically significant difference in this length between patients suffering from diverse ischemic diseases and a healthy control group. Chen et al.^19^ transformed these perfusion distances into perfusion deficit areas by thresholding all pixel that are further away from the next vessel pixel than a pre-defined distance. Afterwards they used these areas to compute the geometric perfusion deficit percentage, which is the total perfusion deficit area divided by a total reference area and showed a statistically significant difference of this metric between diabetic retinopathy patients and healthy controls.

However, all these more sensitive features have never been evaluated for the task of distinguishing glaucoma patients from a healthy control group.

We propose novel features based on the probability density function of the perfusion deficit areas computed from the perfusion distance. We train a support vector machine (SVM) to distinguish between glaucoma patients and a healthy control group and show that it outperforms other handcrafted features like the vessel density, intercapillary areas and the geometric perfusion deficit percentage. Moreover, the results of a convolutional neural network are in the same range as the presented method, making the proposed features a computationally efficient, simple to implement, and explainable alternative.

## Methods

**Figure 1:**
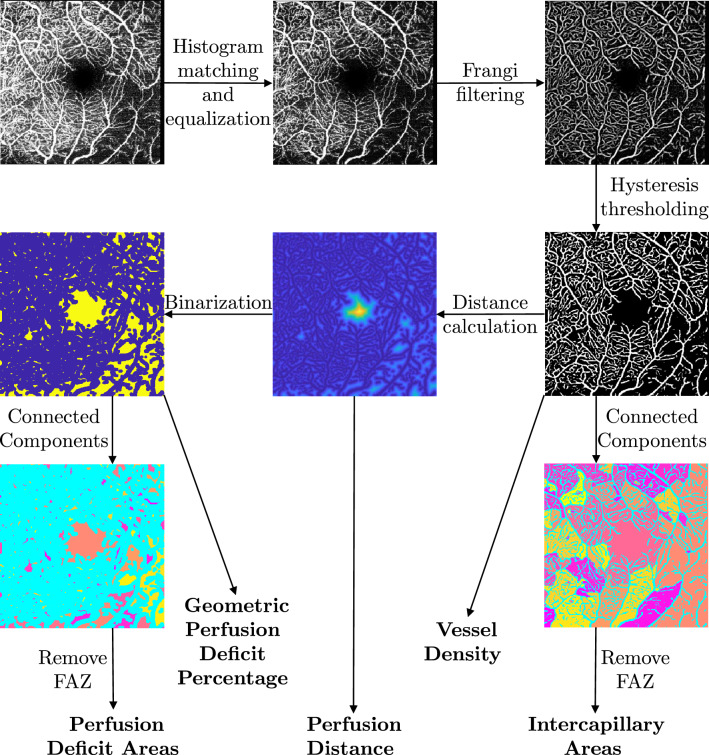
Visualization of the algorithmic workflow. A vessel segmentation is obtained by matching the histogram of the input image to the one of a reference image and equalizing the resulting histogram followed by a Frangi vesselness filter and a subsequent hysteresis thresholding operation. From this segmentation the vessel density can be directly computed as relative frequency. Moreover the intercapillary areas can be obtained by identifying the connected components. For the proposed method, a perfusion distance map is computed from which the perfusion distance values can be obtained. This perfusion distance map is subsequently binarized. From these binarized perfusion deficit areas, the geometric perfusion deficit percentage can be calculated. Moreover, the distinct perfusion deficit areas can be identified using connected components. Information about those regions can be summarized using features of the probability density function of their areas.

Our proposed method computes features of the probability density function of the intercapillary or non-perfused areas based on the perfusion distance instead of the vessel segmentation directly, which makes the approach more robust. An algorithmic overview of the steps and related methods is given in  Fig. 1.

In a first step, the histogram of the input image is matched with the histogram of a reference image. As reference image, a good-quality scan of a healthy volunteer without artifacts was chosen. This compensates for possibly different illumination in the acquisitions and facilitates further processing steps since the same hyperparameters fit better on different scans. Afterwards the image is contrast enhanced using a contrast limiting adaptive histogram equalization (CLAHE). In order to highlight the vessel structure, a Frangi vesselness filter is employed and the image is subsequently binarized using hysteresis thresholding. The vessel segmentation is post-processed by using morpholocial operations to fill small holes and bridge gaps.

From this binary vessel map, the vessel density (VD) can directly be computed as the ratio of vessel pixels to the total sum of pixels in the image. Moreover, the intercapillary or non-perfused areas can be determined. To this end, the image is inverted and the connected components are identified. Each connected component then resembles one intercapillary or non-perfused area.

Our method uses the segmentation to first create a perfusion distance map. It is computed as the Euclidean distance transform of the segmentation using the efficient algorithm of Maurer et al.^20^. Each pixel now has the value of the Euclidean distance to the next vessel pixel and can be used to obtain the perfusion distance values as used by Lauermann et al.^18^.

The resulting perfusion distance map can be binarized by only keeping pixels that have a higher distance than a given threshold and setting all other pixels to zero, leading to the perfusion deficit areas. This binarized map can be used to compute the geometric perfusion deficit area percentage by computing the ratio of the perfusion deficit area pixels to all the pixels in the reference area as described by Chen et al.^19^.

**Figure 2:**
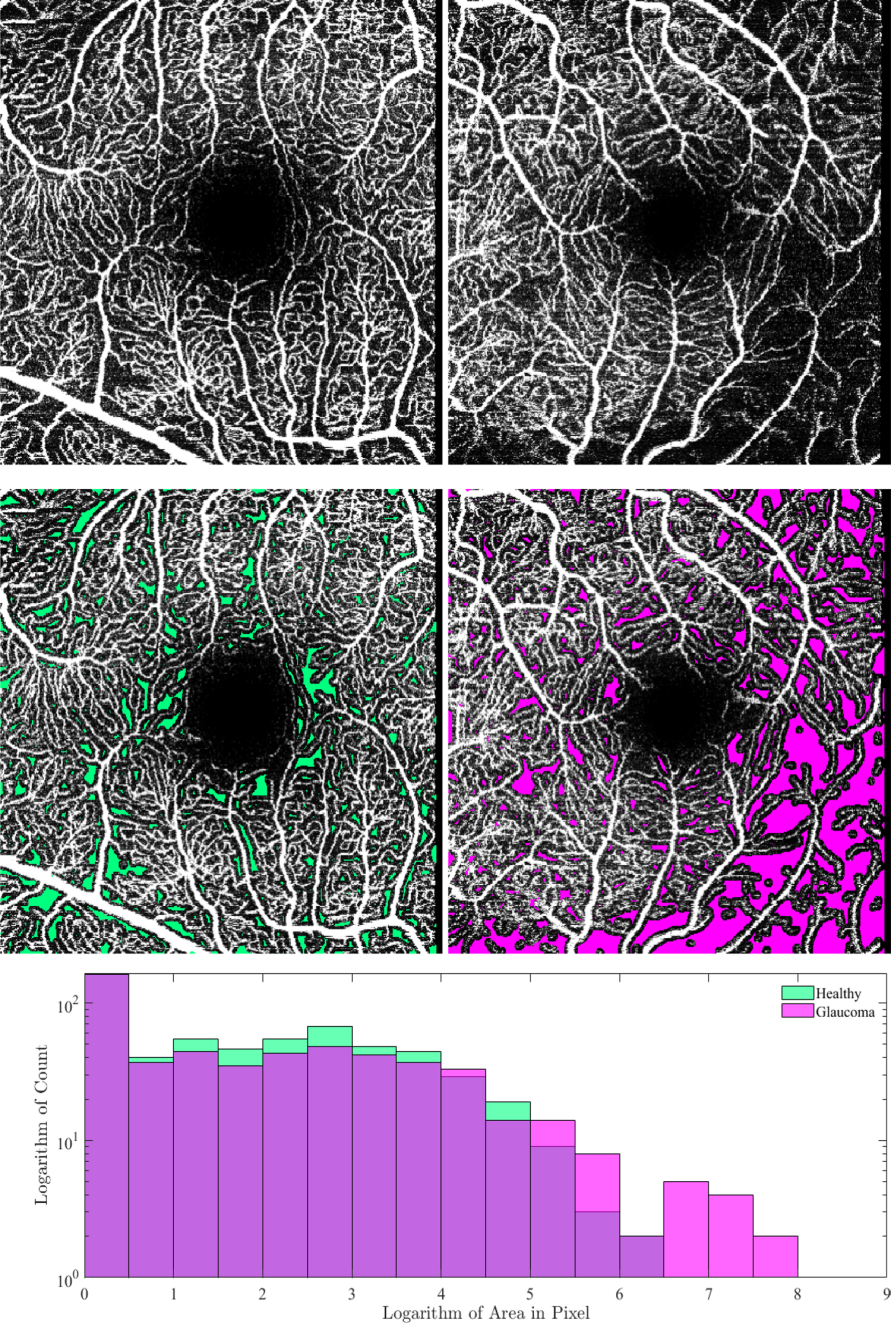
The first row shows the input OCTA image of a healthy control on the left and a glaucoma patient on the right, while the middle row visualizes the corresponding identified perfusion deficit areas overlayed over these scans. The last row presents the histogram of the logarithm of the area of the perfusion deficit areas.

We propose to determine individual perfusion deficit areas, as in the case of the intercapillary areas, by identifying connected components. Again, each connected component contains the information for one perfusion deficit area. We compute the area of these regions and summarize them by a probability density function. Subsequently, this distribution can be represented by features like the maximum, mean, standard deviation or kurtosis. This approach is depicted for a healthy control and a glaucoma patient in Fig. 2.

**Figure 3:**
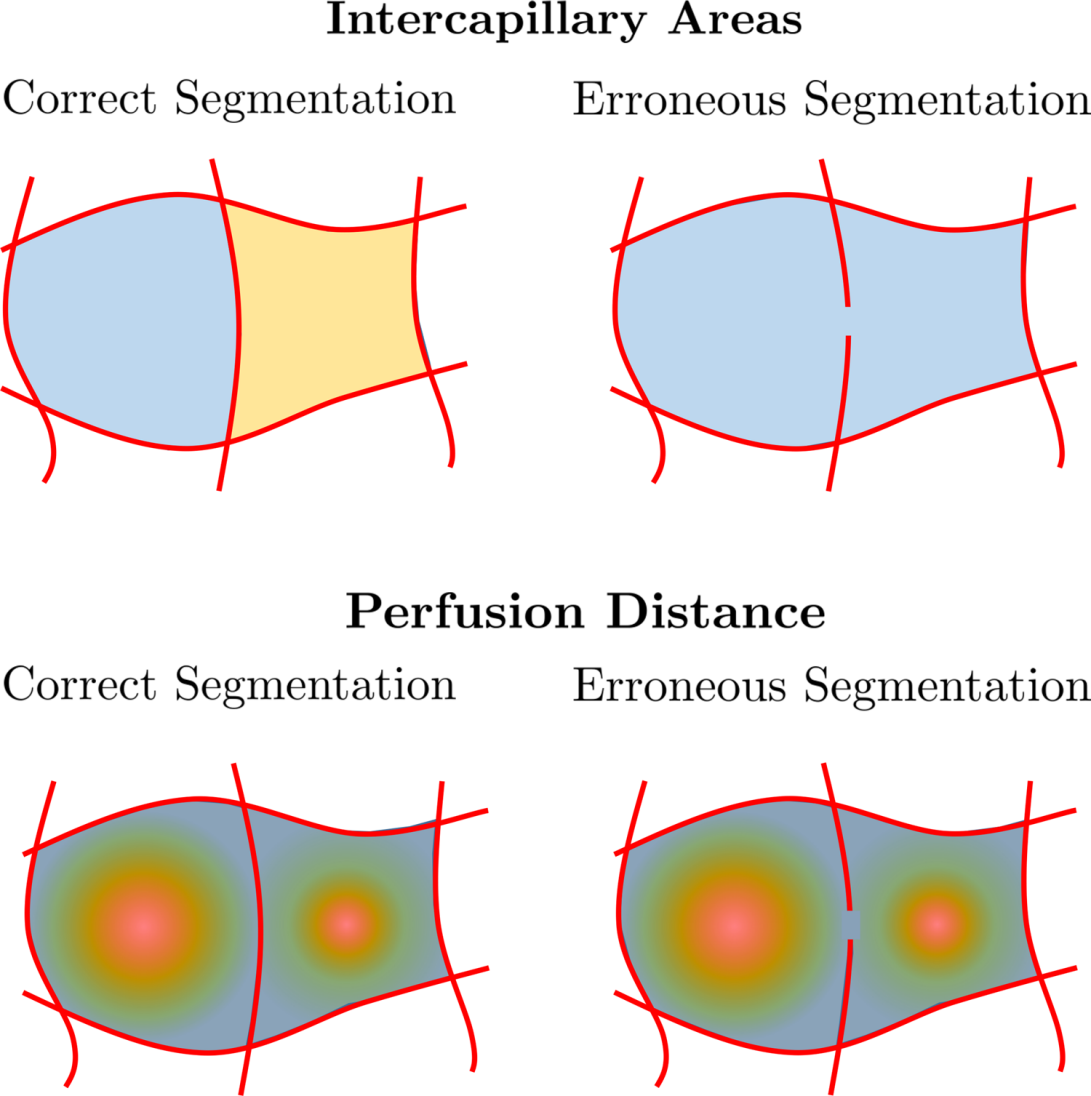
Visualization of the influence of segmentation errors on the intercapillary areas and the perfusion distance. In this example when the segmentation is correct, there are two distinct intercapillary areas. However, even if there are only small errors, both distinct areas merge to one with a much larger area. This will distort the features computed from these areas. In contrast to that, the perfusion distance is barely influenced by small segmentation errors.

The proposed algorithm can be expected to be more sensitive to smaller changes than the vessel density since the perfusion distance areas directly increase when vessels are not visible on OCTA images anymore due to a reduced perfusion. Moreover, by using the perfusion distance map and computing the areas from that, it should simultaneously be more robust than the intercapillary areas computed directly from the vessel segmentation, because the influence of segmentation errors is reduced. This is illustrated in  Fig. 3. Since the proposed features describe the probability density function of area of diffusion deficit to a certain extent, this should also theoretically contain more information than the geometric perfusion deficit percentage, which combines this information into a single value.

## Evaluation

In order to evaluate the performance of the proposed features, they were used to distinguish between glaucoma patients and a healthy control group. The results were compared to methods using the vessel density, the intercapillary areas, the perfusion distances, the geometric perfusion deficit percentage and convolutional neural networks in a five-fold cross validation for four different retinal OCTA projections.

### Data

352 eyes of 244 patients of the Department of Ophthalmology, University of Erlangen-Nürnberg and the Erlanger Glaucoma Registry (Erlangen Glaucoma Registry, ISSN 2191-5008, CS-2011; NTC00494923) were recruited for the present study: 154 eyes from 110 healthy control subjects and 198 eyes from 132 glaucoma patients.

All subjects received a standardized ophthalmological examination including fundus photography, standard white-on-white full-field perimetry (Octopus 500, G1 protocol, Interzeag, Schlieren, Switzerland) and measurement of intraocular pressure (IOP) by Goldmann applanations tonometry. The latter was measured twice and corrected for the central corneal thickness (CCT) according to Kohlhaas et al.^21^. CCT was measured using the central ultrasonic pachymetry (Pachymeter SP-100)).

The patients in each group were selected based on the following inclusion criteria. The control cohort was defined as eyes showing no systemic disease with ophthalmological involvement or ophthalmological dysfunction neither having had any ophthalmic surgery. The glaucoma data consisted of 47 eyes from glaucoma suspects and 151 eyes from glaucoma patients. Glaucoma suspects were defined as having a normal visual field, and an elevated IOP (above 21 mmHg, ocular hypertension, OHT) or showed additiv glaucomatous optic disc damage classified by Jonas et al.^22^ (pre-perimetric glaucoma). Glaucoma patients showed perimetric field defects and alterations of the optic nerve head according to Jonas^22^. This group was further subdivided into 116 eyes from patients having an elevated IOP (above 21 mmHg) and 35 eyes from those not having an increased IOP (normal tension glaucoma). Exclusion criteria were an age below 18 years and any further eye disorders and/or systematic disorders with ocular involvement at the time of enrolement.

From the eyes of the control cohort, 70 were male and 84 were female with an average age of 63,38 ± 13,10 years. The glaucomatous eyes consisted of 104 male and 94 female eyes with an average age of 65,04 ± 11,69 years. The visual field testing yielded a mean defect of $2.04 \pm 1.96 \text { dB}$ and a loss variance of $6.52 \pm 8.60 \text { dB}^{2}$ for the glaucoma suspects, $7.33 \pm 4.71 \text { dB}$ and $50.39 \pm 40.72\text { dB}^{2}$ for the normal tension glaucoma group and of $8.36 \pm 5.89 \text { dB}$ and $38.60 \pm 30.64\text { dB}^{2}$ for the rest.

En face OCTA imaging was done using Heidelberg Spectralis II OCT (Heidelberg, Germany). Images were recorded with a 15×15^∘^ angle and a lateral resolution of 5.7 $$\upmu \hbox {m}$$/pixel, resulting in a retinal section of $$\sim$$$$3 \times 3~\hbox {mm}$$. Acquisitions consisted of 512 A-scans per B-scan and 512 consecutive B-scans in the macular region. OCTA en face projections of the superficial vascular plexus (SVP), the intermediate capillary plexus (ICP), deep capillary plexus (DCP), and the whole retina (retina = SVP + ICP + DCP) were automatically segmented and computed using the manufacturer’s software (Heidelberg Eye Explorer Version 1.12.1.0). All images were visually reviewed by an ophthalmology expert who excluded images that the expert considered to be of insufficient quality for use in clinical routine.

The study has been approved by the ethics committee of the university of Erlangen-Nuremberg and performed in accordance to the tenets of the Declaration of Helsinki. Informed written consent was obtained from each participant.

This data was split into 60% training set, 20% validation set and 20% test set, with all eyes from one patient belonging exclusively to only one set. This leads to a distribution of approximately 211 projections in the training, 71 in the validation and 71 in the test set.

### Experiments

For the vessel density, intercapillary area and perfusion distance methods, features were extracted and a support vector machine (SVM) was trained on these features. The vessel density (VD) and the geometric perfusion deficit percentage (GPDP) are scalar values and were used as the sole inputs to the SVMs. In case of perfusion distances (PD) the maximum, mean, standard deviation and kurtosis of their distribution were computed and these four features used as input to the SVM. For the intercapillary areas (IA) and the perfusion deficit areas (PDA) the area, measured in pixel, for each identified connected component was measured and the same four features as for the PD of their distribution computed and used as input for the SVM. An overview of the different features used per method is given in Table 1. Different SVM hyperparameter settings were trained on the training set and their performance evaluated on the validation set. As a performance measure, the area under the receiver operating characteristics (AUROC) and the F1-score were used. For parameter selection, the SVM setup that yielded the highest AUROC metric on the validation set was chosen for the final evaluation on the test set.

**Table 1:** Overview of the inputs to the classification algorithm used per method in the evaluation of this study.

Approach	Input to classification			
CNN	Image			
VD	Percentage			
GPDP	Percentage			
IA	Maximum	Mean	Standard deviation	Kurtosis
PD	Maximum	Mean	Standard deviation	Kurtosis
PDA	Maximum	Mean	Standard deviation	Kurtosis

*CNN* convolutional neural network, *VD* vessel density, *GPDP* geometric perfusion deficite percentage, *IA* intercapillary areas, *PD* perfusion distance, *PDA* perfusion deficite areas.

For the convolutional neural network (CNN) no features needed to be extracted as the network learns them automatically. As architecture a DenseNet161^23^ and a WideResNet-101-2^24^ were chosen for the retina, SVP, DCP and the ICP projection respectively since Schottenhamml et al.^13^ evaluated in their paper that these architectures performed best from their evaluated architectures on $3 \times 3~\hbox {mm}$ en face projections acquired with a Heidelberg Spectralis II OCT. Also the training parameters were chosen as described in their paper. As for the other approaches, the network was first trained on the training set and then evaluated on the validation and test sets with the AUROC as performance metric.

In this study, a five-fold cross validation was performed, where it was made sure that the eyes of one patient exclusively belonged to one set and were not split across sets. Consequently, each eye belonged once to the validation and once to the test set. Moreover, each capillary plexus (SVP, ICP and DCP) and the retina projection (Retina) were evaluated separately since they can be affected differently by the disease.

## Results

The performance, as measured with the AUROC and F1-scores, for each fold and the mean over all folds of the five-fold cross validation for the different methods and plexuses on the test set are shown in  Table 2. It shows that for most plexuses, the CNN shows the best performance closely followed by our proposed approach using the perfusion deficite areas. In the ICP our proposed method yields even higher AUROC and F1-scores. However, our proposed method has the highest scores among the handcrafted features. The worst performance over all plexuses is shown by the intercapillary areas approach, followed by the vessel density. Also the F1-scores show for most cases the same tendency as the AUROC values.

**Table 2:** AUROC values and F1-scores for each fold and the mean over all folds of the five-fold cross validation for the different methods.

Plexus	Method	Fold1		Fold2		Fold3		Fold4		Fold5		Mean	
		AUROC	F1	AUROC	F1	AUROC	F1	AUROC	F1	AUROC	F1	AUROC	F1
Retina	CNN	0.89	0.86	0.93	0.86	0.78	0.68	0.95	0.86	0.85	0.72	0.88	0.79
	**PDA**	**0.86**	**0.82**	**0.92**	**0.83**	**0.72**	**0.62**	**0.91**	**0.83**	**0.87**	**0.73**	**0.86**	**0.766**
	GPDP	0.85	0.77	0.89	0.76	0.61	0.59	0.85	0.76	0.85	0.75	0.81	0.726
	PD	0.85	0.79	0.89	0.83	0.60	0.56	0.86	0.76	0.85	0.80	0.81	0.748
	VD	0.76	0.69	0.74	0.65	0.57	0.56	0.83	0.75	0.78	0.69	0.74	0.668
	IA	0.67	0.63	0.58	0.58	0.58	0.51	0.66	0.56	0.69	0.58	0.64	0.572
SVP	CNN	0.96	0.85	0.92	0.82	0.90	0.79	0.94	0.89	0.95	0.86	0.93	0.842
	**PDA**	**0.93**	**0.89**	**0.90**	**0.81**	**0.86**	**0.78**	**0.94**	**0.87**	**0.92**	**0.82**	**0.91**	**0.834**
	PD	0.91	0.86	0.90	0.86	0.81	0.73	0.93	0.83	0.89	0.75	0.89	0.806
	GPDP	0.88	0.79	0.91	0.80	0.75	0.65	0.92	0.79	0.89	0.79	0.87	0.764
	VD	0.83	0.70	0.85	0.72	0.76	0.72	0.92	0.87	0.85	0.76	0.84	0.754
	IA	0.76	0.77	0.88	0.79	0.80	0.73	0.82	0.77	0.84	0.70	0.82	0.752
ICP	**PDA**	**0.83**	**0.75**	**0.90**	**0.75**	**0.85**	**0.70**	**0.92**	**0.83**	**0.89**	**0.79**	**0.88**	**0.764**
	CNN	0.87	0.73	0.90	0.75	0.76	0.68	0.90	0.79	0.89	0.80	0.86	0.750
	PD	0.84	0.76	0.91	0.74	0.72	0.70	0.92	0.82	0.87	0.77	0.85	0.758
	GPDP	0.83	0.73	0.89	0.72	0.73	0.68	0.90	0.79	0.81	0.72	0.83	0.728
	VD	0.73	0.70	0.78	0.75	0.66	0.61	0.90	0.82	0.81	0.72	0.78	0.720
	IA	0.64	0.58	0.52	0.55	0.66	0.63	0.77	0.69	0.58	0.49	0.63	0.588
DCP	CNN	0.82	0.70	0.97	0.87	0.82	0.75	0.92	0.86	0.82	0.72	0.87	0.780
	**PDA**	**0.80**	**0.75**	**0.94**	**0.87**	**0.71**	**0.68**	**0.88**	**0.82**	**0.75**	**0.70**	**0.82**	**0.764**
	GPDP	0.73	0.63	0.93	0.79	0.64	0.59	0.90	0.83	0.71	0.62	0.78	0.692
	PD	0.80	0.73	0.86	0.79	0.65	0.61	0.84	0.73	0.71	0.69	0.77	0.717
	VD	0.60	0.59	0.82	0.66	0.64	0.61	0.80	0.72	0.61	0.61	0.70	0.638
	IA	0.52	0.48	0.62	0.59	0.57	0.48	0.57	0.51	0.53	0.48	0.562	0.508

For each plexus the methods are sorted according to their mean AUROC performance in descending order. The proposed features are highlighted in bold.

(*CNN* convolutional neural network, *PDA* perfusion deficite areas, *GPDP* geometric perfusion deficite percentage, *PD* perfusion distance, *VD* vessel density, *IA* intercapillary areas) and plexuses (*Retina* retina, *SVP* superficial vascular plexus, *ICP* intermediate capillary plexus, *DCP* deep capillary plexus) on the test set.

## Discussion

The weak performance of the intercapillary areas approach can be explained by limitations of the segmentation algorithm when applied to our data. As can be seen in  Fig. 1 the segmentation of the vascular network shows several gaps and the resulting non-perfused areas are very large, combining regions that are very likely multiple intercapillary areas in reality. Consequently the features computed from these areas are distorted and do not reflect pathologic changes very well. These results demonstrate the low robustness of the algorithm and the need for a very accurate segmentation in order to yield meaningful results. The segmentation algorithm used in this paper is rather basic and the segmentation results can probably be improved by using e.g. neural networks to perform this task. This however, would lead to a need for sufficient training data for a segmentation network which is very time-consuming and labour-intensive. Moreover, even though the segmentation can be improved, even neural networks will likely not provide a sufficiently accurate segmentation. So the problem with errors in the segmentation could be reduced but not completely eliminated.

The results of the SVM trained on the vessel density are in the same range as reported in the paper by Schottenhamml et al.^13^. This approach is able to detect changes between glaucoma patients and the healthy control group, however it is not very sensitive to smaller changes in early stages of the disease. But it is more robust and less influenced by segmentation errors than the intercapillary areas, leading to a better performance than the IA approach. Since the vessel density is the fraction of vessel pixel in a projection and it can be assumed that en face projections from healthy controls are affected in the same way as projections from glaucoma patients by segmentation errors, the overall performance of this feature will probably not increase much with a better segmentation.

The SVMs trained on the perfusion distance features and the geometric perfusion deficit percentage show similar results and occupy the third and fourth places. They are performing better than the aforementioned features and are apparently more sensitive to smaller changes in the pathology while being more robust to segmentation errors. This is to be expected as explained in  Fig. 1. However, both approaches have so far only been evaluated for distinguishing ischemic retinal diseases (diabetic retinopathy, central and branch retinal vein occlusion, hypertensive retinopathy, and occlusive retinal vasculitis in sarcoidosis for the perfusion distances and diabetic retinopathy for the geometric perfusion deficit percentage) to a healthy control cohort and never for the task of distinguishing glaucoma patients from the healthy control cohort. Consequently the results from this study show, that both also provide acceptable biomarkers for the latter task.

Using the features of the area probability density function of the perfusion deficit areas outperforms the other feature-based machine learning approaches. This indicates that it is more sensitive to a decreased perfusion and can also pick up cases with subtler changes. Moreover, it apparently also captures more information than the perfusion distances and the geometric perfusion deficit percentage, both methods which also rely on the more robust perfusion deficit area segmentation. For the geometric perfusion deficit percentage this is not surprising, since this approach relies on the same information but condenses them into a single scalar value while the method presented in this study uses multiple features.

**Figure 4:**
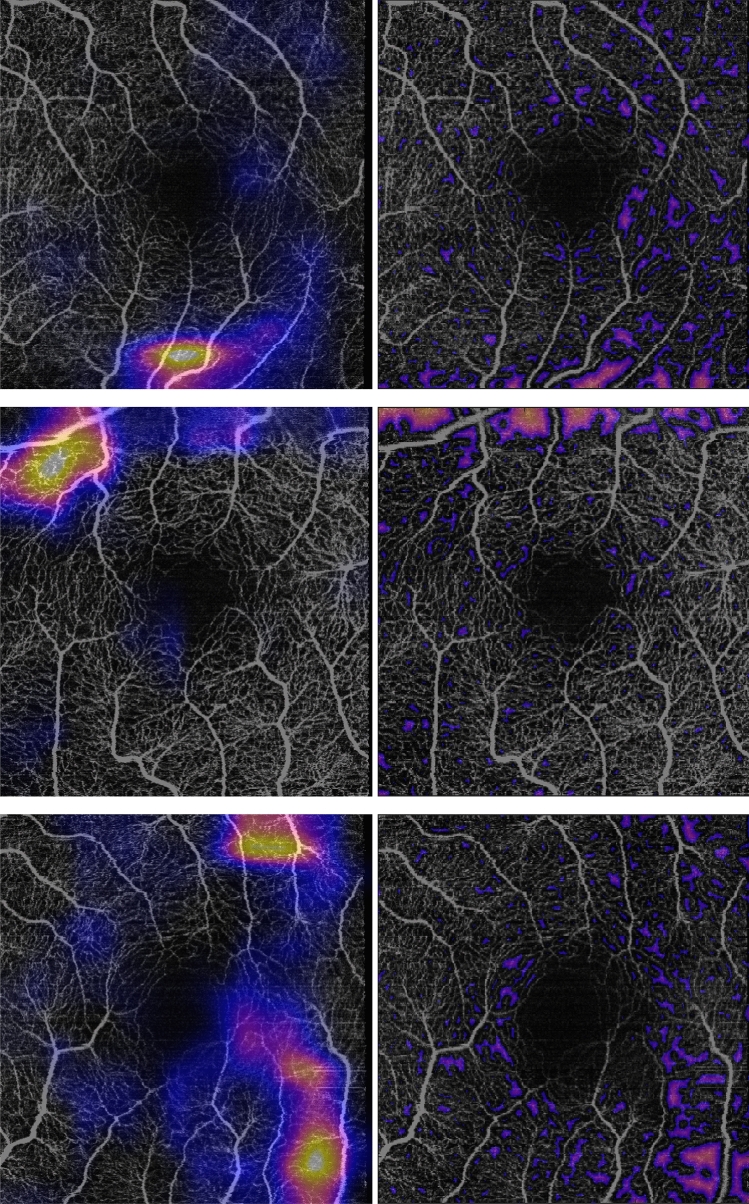
Visual examples for the regions highlighted by the Grad-CAM algorithm (left column) and the corresponding thresholded perfusion distance maps (right column) for glaucoma patients showing a close correspondence between the two methods.

The CNN performs best which is not surprising as in many medical fields the deep learning methods are reported to outperform traditional machine learning approaches. However, neural networks are computationally more expensive and more complicated to implement and understand. Moreover the previous study suggests that the CNNs also seem to focus on the non-perfused regions. Consequently, the intention of this study was to investigate biomarkers that capture the same regions of interest as the previously reported convolutional neural network and therefore obtain a similar performance while being computationally more efficient, simple to implement and more explainable in comparison to the black box approach where the internal decision making is not comprehensible for humans.  Figure 4 shows visual examples for regions highlighted by the Grad-CAM algorithm applied to the CNN and the thresholded perfusion distance maps of glaucoma patients. This comparison shows a close correspondence between the two approaches. Moreover, the results in  Table 2 obtained by the CNN and the perfusion deficit area approach presented in this study are in the same ballpark. Consequently, the proposed features can be an explainable alternative, since the performance is not much lower than the deep learning approach. This is still very relevant if the method is used as a tool for clinicians to support them in their decision-making process since it offers a clearer reasoning about the decision.

The features have also been evaluated on different plexuses and the retina projection. This makes sense since in the retina projection, larger non-perfused areas in one plexus can be partitioned into multiple by vessels from other plexuses at the same position. Consequently if one plexus is more affected by the disease than others this can be hidden when using the projection over all plexuses. When looking at the performance of the different approaches across the different plexuses they resemble the ones previously published. The SVP seems to be the best suited plexus for the task of distinguishing glaucoma patients from the healthy control cohort.

So far only the areas of the perfusion deficit areas has been used for the probability density function. An interesting future direction is to investigate whether features describing the shape instead of the area of regions of perfusion deficit provides additional information.

Moreover, in this study only the distinction between a healthy control group and glaucoma patients was considered. So further studies are necessary to evaluate this novel approach for different glaucoma subtypes to see from which level of glaucoma damage onwards a distinction between glaucoma patients and the healthy controls can be detected. However, more data from these subtypes needs to be acquired in order to enable a reasonable training and evaluation.

## Summary and conclusion

In this study we have presented and evaluated new features based on the probability density function of the perfusion deficit areas and evaluated their performance with existing features from literature and convolutional neural networks. The evaluation showed that it is more robust and sensitive than existing handcrafted features when distinguishing between glaucoma patients and healthy controls. While there is a small remaining gap compared to the performance of the best evaluated deep learning models, they are computationally more efficient, simple to implement and more explainable and therefore more useful in supporting clinical decision-making.

## Data Availability

The dataset analysed during the current study is not publicly available due to general data privacy regulations but is available from the corresponding author on reasonable request.
